# Gilteritinib as Bridging and Posttransplant Maintenance for Relapsed Acute Myeloid Leukemia with FLT3-ITD Mutation Accompanied by Extramedullary Disease in Elderly

**DOI:** 10.1155/2023/7164742

**Published:** 2023-08-23

**Authors:** Masuho Saburi, Masanori Sakata, Rika Maruyama, Yosuke Kodama, Hiroyuki Takata, Yasuhiko Miyazaki, Katsuya Kawano, Junpei Wada, Shogo Urabe, Eiichi Ohtsuka

**Affiliations:** ^1^Department of Hematology, Oita Prefectural Hospital, Oita, Japan; ^2^Department of Clinical Laboratory Technology, Oita Prefectural Hospital, Oita, Japan; ^3^Department of Pathology, Oita Prefectural Hospital, Oita, Japan

## Abstract

A 69-year-old woman was diagnosed with acute myeloid leukemia (AML) with an FMS-like tyrosine kinase 3-internal tandem duplication (FLT3-ITD) mutation. Complete remission (CR) was achieved after induction therapy, but AML resulted in a hematological relapse two months after the consolidation chemotherapy. Relapse was accompanied by multiple skin lesions that demonstrated leukemic cell infiltration as well as a drooping right eyelid with extroversion of the eye due to right oculomotor palsy. Gilteritinib was started as salvage therapy, and bone marrow blasts decreased to 0.8% after one month. Two months later, the eye symptoms improved, and the patient underwent cord blood transplantation (CBT). The skin lesions disappeared after the conditioning regimen, and the patient achieved CR status with complete donor chimerism at day 28. Gilteritinib was restarted as posttransplant maintenance therapy on day 53 of CBT. No adverse events other than mild hepatotoxicity were observed, and the patient was alive and in CR status, while continuing gilteritinib at one year and seven months after CBT. Bridging and posttransplant maintenance therapy with gilteritinib may be a promising therapeutic option for relapsed AML with the FLT3-ITD mutation in elderly patients.

## 1. Introduction

FMS-like tyrosine kinase 3 (FLT3) mutations are found in approximately 30% of all acute myeloid leukemia (AML) patients as either FLT3-tyrosine kinase domain (FLT3-TKD) or FLT3 internal tandem duplication (FLT3-ITD) mutations. FLT3-TKD and FLT3-ITD mutations cause uncontrolled signaling through ERK signaling and PI3-kinase signaling. In the case of FLT3-ITD mutation, there is also activation of STAT5 signaling that drives stem cell transformation. AML with FLT3-ITD mutation is associated with a poor clinical outcome characterized by a high relapse rate with rapid progression, despite a temporary response to conventional chemotherapy [1]. The FLT3-tyrosine kinase inhibitors (TKI), gilteritinib [2] and quizartinib [3], have been developed for the treatment of AML with FLT3-ITD mutation and have shown greater efficacy and longer survival compared with conventional salvage chemotherapy. However, the outcomes of relapsed AML with FLT3-ITD mutation are inadequate in FLT3-TKI monotherapy, and stem cell transplantation is indicated for transplant-eligible patients. We report the case of an elderly patient with relapsed AML with FLT3-ITD mutation accompanied by extramedullary disease, who received reinduction therapy using gilteritinib followed by cord blood transplantation (CBT), and posttransplant maintenance therapy also using gilteritinib.

## 2. Case Presentation

**Figure 1:**
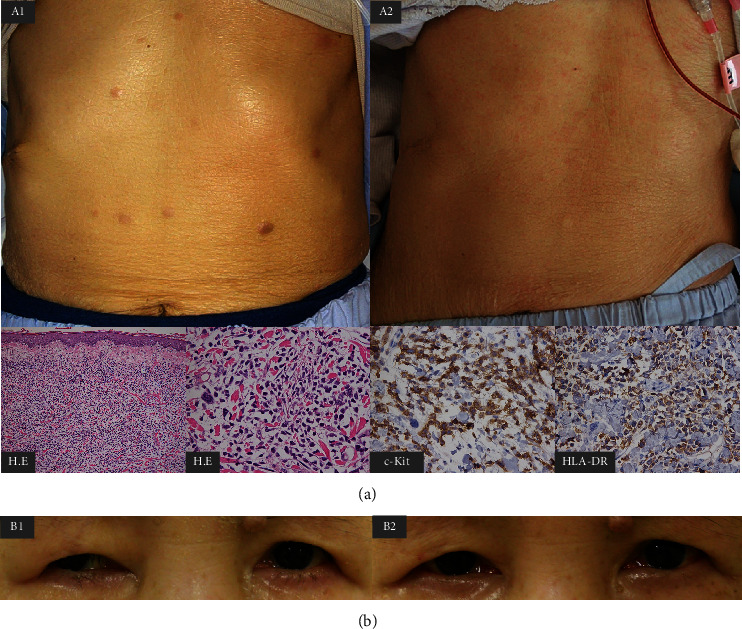
Macroscopic and histological findings of skin lesions with infiltration of leukemic cells at relapse (hematoxylin-eosin staining and immunostaining) and macroscopic findings on day 14 of cord blood transplantation (a). Scattered eruptive skin lesions are observed in the abdomen (A1). Pathologically, there is diffuse proliferation of leukemic cells, with positivity for c-kit and HLA-DR. Skin lesions disappeared after the conditioning regimen, and a rash due to acute graft-versus-host disease developed on day 14 of cord blood transplantation (A2). Macroscopic findings of drooping of the right eyelid with extroversion of the eye due to right oculomotor palsy (b) at relapse (B1) and at one month after cord blood transplantation (B2).

**Figure 2:**
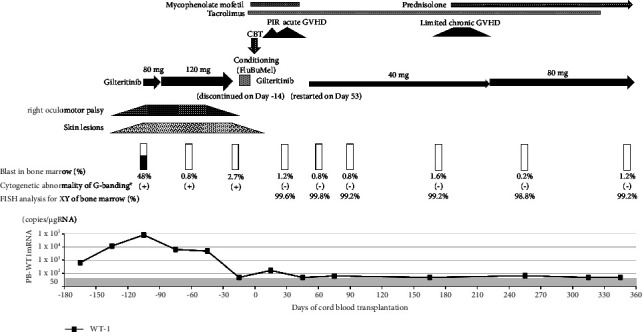
Clinical course following treatment with gilteritinib. ^*∗*^Cytogenetic abnormality by G-banding: 46, XX, t(1; 17)(p34; q21.2). CBT, cord blood transplantation; FluBuMel, fludarabine/busulfan/melphalan; PIR, pre-engraftment immune reaction; GVHD, graft-versus-host disease; WT1, Wilms' tumor 1; FISH, fluorescence in situ hybridization.

A 69-year-old woman presented to our hospital with fever and cough. A complete blood cell analysis showed white blood cell count of 140,000/L (blast cells 80%), platelet count of 21.8 × 10^4^/L, and hemoglobin concentration of 10.5 g/dL with elevated serum concentration of lactate dehydrogenase (894 U/L). Bone marrow aspiration revealed numerous blast cells without maturation (90.2%), and 93% of blast cells were positive for myeloperoxidase. Reverse transcriptase polymerase-chain reaction yielded negative results for recurrent genetic abnormalities. G-banding showed that 46, XX, t(1; 17)(p34; q21.2), and FLT3-ITD mutation was positive. Wilms' tumor 1 (WT1)-mRNA in peripheral blood was 110,000 copies/*µ*g RNA. Based on these findings, the patient was diagnosed with AML, not otherwise specified (AML without maturation) with FLT3-ITD mutation. Remission induction therapy comprised idarubicin and cytarabine, and first complete remission (CR) was achieved with WT1-mRNA negativity in peripheral blood. As the patient did not wish to receive allogeneic hematopoietic stem cell transplantation at the first remission, consolidation chemotherapies comprising three courses of cytarabine plus anthracycline and A-triple-V (cytarabine, vincristine, vindesine, and etoposide) were completed. Two months after consolidation therapy, leukemia relapsed (blast cells in bone marrow 48% and WT1-mRNA in peripheral blood 87,000 copies/*µ*g RNA), accompanied by multiple skin lesions that demonstrated leukemic cell infiltration (Figure 1(a)). Drooping of the right eyelid was also observed with extroversion of the eye due to right oculomotor palsy (Figure 1(b)). There was no infiltration of leukemic cells in cerebrospinal fluid, and magnetic resonance imaging showed no obvious lesions in the oculomotor or central nerves. We confirmed FLT3-ITD positivity in a companion diagnostic test at relapse. Gilteritinib was started at 80 mg/day and increased to 120 mg/day after one week. Blast cells of bone marrow decreased to 0.8% one month after starting gilteritinib, but pancytopenia persisted along with hypoplastic bone marrow. The skin lesions began to show thinning and no new lesions appeared. The eye symptoms improved two months after starting gilteritinib, and the patient then underwent CBT (Figure 2). Before CBT, G-banding in bone marrow detected 46, XX, and t(1; 17)(p34; q21.2) in 6/20 cells, and the WT1-mRNA level in peripheral blood remained high (6000 copies/*µ*g RNA). Gilteritinib was discontinued one week before starting a conditioning regimen comprising fludarabine (150 mg/m^2^), busulfan (12.4 mg/kg), and melphalan (80 mg/m^2^). The patient received cord blood from a human leukocyte antigen (HLA) 4/6 matched-unrelated male donor, with nucleated cells of 2.31 × 10^7^/kg and CD34-positive cell count of 1.07 × 10^5^/kg. Tacrolimus and mycophenolate mofetil were administered for graft-versus-host disease (GVHD) prophylaxis. Skin lesions disappeared after the conditioning regimen. Pre-engraftment immune reaction and acute GVHD grade 1 (skin stage 1) developed on days 9 and 14, respectively, and improved only with appropriate administration of hydrocortisone and topical steroid. Engraftment was achieved (neutrophils on day 16, platelets on day 30), and bone marrow examination on day 28 confirmed CR. Complete donor chimerism was confirmed for T-cells by short tandem repeat of peripheral blood and bone marrow by XY fluorescence in situ hybridization. WT1-mRNA in peripheral blood became undetectable on day 28. Gilteritinib 40 mg/day was restarted as post-transplant maintenance therapy on day 53 (neutrophil count 2000/*µ*L and platelet count 52,000/*µ*L). Mild hepatotoxicity was observed (grade 2 according to the National Cancer Institute Common Terminology Criteria for Adverse Events (CTCAEs)) that was resolved spontaneously in one month without discontinuation of gilteritinib. There were no other adverse events such as bone marrow suppression, QTc prolongation, or diarrhea. The patient developed chronic GVHD (lichen planus-like oral mucosa, arthralgia, and hypoalbuminemia) on day 187, following dose reduction of tacrolimus. Chronic GVHD was relieved by adding prednisolone 0.5 mg/kg to tacrolimus. Gilteritinib was increased to 80 mg/day from day 228, and mild hepatotoxicity (CTCAEs grade 2) was observed with gradual improvement in grade 1 without discontinuation of gilteritinib. The patient is currently alive in CR status with continuing gilteritinib 80 mg/day at one year and seven months after transplantation.

## 3. Discussion

In the present case, relapsed AML with FLT3-ITD mutation accompanied by extramedullary disease in an elderly patient was treated by the reinduction therapy using gilteritinib followed by CBT with posttransplant maintenance gilteritinib therapy. The known prognostic factors for relapsed AML are age, transplantation in first remission, time to relapse, chromosomal karyotype, and the presence of FLT3-ITD mutation [4]. Allogeneic transplantation can be considered in second CR, but only 27% of patients in the high-risk group, including AML with FLT3-ITD mutation, are reported to achieve second CR by chemotherapy [5]. In addition, it is extremely important to achieve second CR by a tolerable therapy in order to decrease nonrelapse mortality (NRM) following stem cell transplantation in elderly patients. In elderly patients with early relapse after consolidation chemotherapy, conventional salvage chemotherapies may result in severe myelosuppression, infection, and high risk of cardiomyopathy due to anthracycline accumulation toxicity. Bridging and post-transplant maintenance therapy with FLT3-TKI may be important to reduce NRM and maintain long-term remission after transplantation for relapsed AML with FLT3-ITD mutation in elderly patients. In the ADMIRAL trial, there was no mention about data of HSCT in patients ≥65 years old in gilteritinib arm [2]. In the post hoc analysis of the ADMIRAL trial [6], there were only four patients ≥65 years old, who were alive after two years in gilteritinib arm, and they were not described with or without HSCT. Thus, there are no previous data of this therapeutic approach in elderly patients.

Gilteritinib is an FLT3/AXL inhibitor that has inhibitory activity against both the FLT3-ITD and FLT3-TKD mutations. In comparison with the conventional salvage chemotherapy in ADMIRAL trial, treatment with gilteritinib resulted in a significantly higher response rate and longer overall survival; in addition, a higher percentage of patients underwent transplantation in relapsed/refractory AML with FLT3 mutations [2]. Adverse events of grade 3 or higher occurred less frequently in the gilteritinib group than in the chemotherapy group. This result suggests the utility of gilteritinib for bridging to transplantation in elderly patients.

Regarding maintenance therapy with FLT3 inhibitors after transplantation [7], sorafenib, the first-generation FLT3-TKI, was shown to prolong the relapse-free survival in the SORMARINE trail [8] and in a phase 3 study in China [9]. The post hoc analysis of the ADMIRAL trial showed a significantly longer median overall survival in patients with gilteritinib after transplantation compared to patients without gilteritinib after transplantation [6]. The efficacy of maintenance therapy after transplantation using gilteritinib is currently being evaluated in a phase 3 trial (MORPHO trial and NCT02997202). The present elderly patient achieved CR with gilteritinib followed by CBT without serious complications, and gilteritinib was restarted as a maintenance therapy two months after CBT. No adverse events other than mild hepatotoxicity were observed and the patient was alive in CR status at one year and seven months after CBT. Although skin lesions and oculomotor palsy developed at hematological relapse, the lesions thinned and no new lesions appeared, following gilteritinib administration, finally disappearing after the conditioning regimen. This clinical course suggests that gilteritinib as well as conditioning regimen following CBT may be effective on skin lesions. Regarding the oculomotor palsy, no leukemic cell infiltration was detected pathologically or radiologically, but the improvement in the clinical course after starting gilteritinib suggests that the eye symptoms might have been caused by AML. A previous report of the efficacy of gilteritinib for extramedullary relapse after transplantation [10] supports the efficacy of gilteritinib for extramedullary lesions in the skin and peripheral nerves, as in the present case. This is the first case report to describe the efficacy of gilteritinib for bridging and posttransplant maintenance in relapsed AML with FLT3-ITD mutation accompanied by extramedullary disease in an elderly patient.

A significant limitation in this case is the short observation period. A longer duration is needed to evaluate the efficacy and safety of gilteritinib as posttransplant maintenance therapy. The other limitation was no available data regarding the signal ratio of FLT3-ITD in the companion diagnosis. Bridging and posttransplant maintenance therapy with gilteritinib shows great promise as a therapeutic option, particularly for relapsed AML with FLT3-ITD mutation in elderly patients.

## Data Availability

The data used to support the findings of this case report are included within the article.
